# Towards machine-learning-based on-the-fly analysis of neutron reflectometry

**DOI:** 10.1107/S1600576726002657

**Published:** 2026-05-14

**Authors:** Anne Rentzsch, Valentin Munteanu, Oliver Odira Anyanor, Shreya Shah, Philipp Gutfreund, Rémi Perenon, Anthony Martin Higgins, Vladimir Starostin, Alexander Hinderhofer, Dmitry Lapkin, Frank Schreiber

**Affiliations:** a Institut für Angewandte Physik, Universität Tübingen, 72076Tübingen, Germany; b School of Engineering and Applied Science, Swansea University, SwanseaSA1 8EN, United Kingdom; c Institut Laue–Langevin, 38000Grenoble, France; d Cluster of Excellence ‘Machine learning – new perspectives for science’, Universität Tübingen, Maria-von-Linden-Straße 6, 72076Tübingen, Germany; Universität Duisburg-Essen, Germany

**Keywords:** neutron reflectometry, machine learning, online data analysis, experiment automation

## Abstract

Reflectometry experiments can benefit substantially from recent advances in machine learning, enabling real-time data analysis, informed decision making during measurements, optimized experimental conditions and ultimately closed-loop experimental workflows. While most prior automation efforts have focused on X-ray reflectometry, neutron reflectometry can also benefit to the same extent. We report the first machine-learning-based pipeline for real-time neutron reflectometry deployed at the Institut Laue–Langevin (ILL), Grenoble, France. The system integrates the *reflectorch* package into the data acquisition workflow using the IT infrastructure of the facility. This enables analysis that is two orders of magnitude faster than conventional tools, allowing real-time estimation of physical parameters with associated uncertainties and feedback through a graphical user interface. The pipeline has been successfully tested at the ILL, and can be adapted to work at other neutron facilities seeking to enable real-time feedback-driven reflectometry analysis.

## Abstract 2

An automated machine-learning-based processing and analysis workflow for neutron reflectometry data is presented. The approach has been successfully used for on-the-fly analysis during an experiment at the Institut Laue–Langevin (Grenoble, France) and can be adapted to work at other neutron facilities seeking to enable real-time feedback-driven reflectometry analysis.

## 1. Introduction

Machine learning (ML) tools hold the promise of revolution­izing science in general, including the way experiments are performed and analysed. This also applies to the area of neutron and X-ray scattering (Chen *et al.*, 2021 ▸). The successful development and deployment of certain tools have already been reported for data analysis and for controlling experiments, *e.g.* for smarter scanning (Teixeira Parente *et al.*, 2023 ▸). A very exciting, but also challenging, prospect consists in connecting ML-based data analysis with the control setup of an experiment, which could ultimately be employed for closed-loop experiments (Pithan *et al.*, 2023 ▸). A basis for this is the demonstration of ‘on-the-fly’ data analysis, which processes the recorded data directly during the experiment.

We present here a working ML analysis pipeline for neutron reflectometry (NR). Reflectometry is one of the established techniques in neutron scattering, which can be found at essentially all neutron sources (Sinha, 1991 ▸; Zabel, 1994 ▸; Majkrzak *et al.*, 1998 ▸; Tolan, 1999 ▸; Masoudi & Pazirandeh, 2005 ▸; Daillant & Gibaud, 2009 ▸; Heinrich *et al.*, 2020 ▸). The synergy with X-ray reflectometry (XRR) lies in the fact that it follows exactly the same general formalism in the data analysis process except for the obvious change of using different scattering cross sections. Importantly, the calculation of the full theoretical solution of a reflectometry data set based on a given layered structure, characterized by a scattering length density (SLD) profile ρ(*z*) as a function of the vertical coordinate *z*, must account for multiple scattering effects and uses an iterative process (Parratt, 1954 ▸) or a matrix method (Abeles, 1950 ▸). These calculations form the basis for fitting algorithms which aim to extract the physical parameters of the system from the measured data.

While conventional software fitting solutions for extracting values for the physical parameters from the measured data are available (Nelson & Prescott, 2019 ▸), the very nature of the problem, including the need to provide suitable starting parameters for the fit, makes its solution inherently somewhat slow, so that ML provides an opportunity for efficiency enhancement. Several ML approaches have indeed already been proposed (Greco *et al.*, 2019 ▸; Greco *et al.*, 2021 ▸; Greco *et al.*, 2022 ▸; Hinderhofer *et al.*, 2023 ▸; Pithan *et al.*, 2023 ▸; Munteanu *et al.*, 2024 ▸; Schumi-Mareček *et al.*, 2024 ▸; Starostin *et al.*, 2025 ▸); nonetheless, there remains room for improvement.

In view of existing ML solutions for reflectometry analysis, the challenge is in making the ML solutions available to the facility user. Here we report on the first efforts to establish an ML data analysis pipeline for NR at the Institut Laue–Langevin (ILL), Grenoble, France.

The ML solution itself is based on previous work (Munteanu *et al.*, 2024 ▸). To demonstrate the performance of the newly established analysis pipeline, we studied two thin-film bilayer systems: a model amorphous [fullerene/polystyrene (PS)] system, which has been investigated previously using NR to examine equilibrium and non-equilibrium behaviour (Higgins *et al.*, 2023 ▸; Higgins *et al.*, 2024 ▸), and a model non-fullerene/PS bilayer. The aim here is to follow the mixing of the polymers and small molecules *in situ* as a function of time during thermal annealing of the bilayers. The small molecules are representative of fullerene and non-fullerene electron acceptors used in organic photovoltaics. The mixing of such small molecules within amorphous polymer-rich domains, the proximity of the domain compositions to those of the coexisting compositions at thermodynamic equilibrium and the widths of the interfaces between neighbouring domains (Alqahtani *et al.*, 2021 ▸) can have a significant impact on device efficiency and stability (Li *et al.*, 2017 ▸; Ye *et al.*, 2018 ▸). Significant mixing of the components is expected in both systems. Previous work on fullerene/PS bilayers has found convergence to equilibrium states, consisting of a PS-rich top layer and a pure fullerene bottom layer, given a sufficiently high annealing temperature (Higgins *et al.*, 2023 ▸). After annealing at sufficiently high temperatures, these states have temperature-dependent coexisting compositions (dis­playing upper-critical-solution-temperature-like behaviour) which can be accessed reversibly. Annealing at lower temperatures can, however, lead to significant hysteresis in the layer thicknesses, the layer compositions and the width of the buried (fullerene/PS-rich) interface (Higgins *et al.*, 2024 ▸).

A key technical challenge in the context of real-time analysis is integrating the existing ML solution such that the communication with the data recording, processing and computing infrastructure of the facility, the ILL in this case, works without delays and risks to data safety. We will describe our solution to these challenges in the following. After this general introduction, we will present a description of the IT infrastructure, including a brief description of the adopted ML solution.

## 2. Software architecture

**Figure 1:**
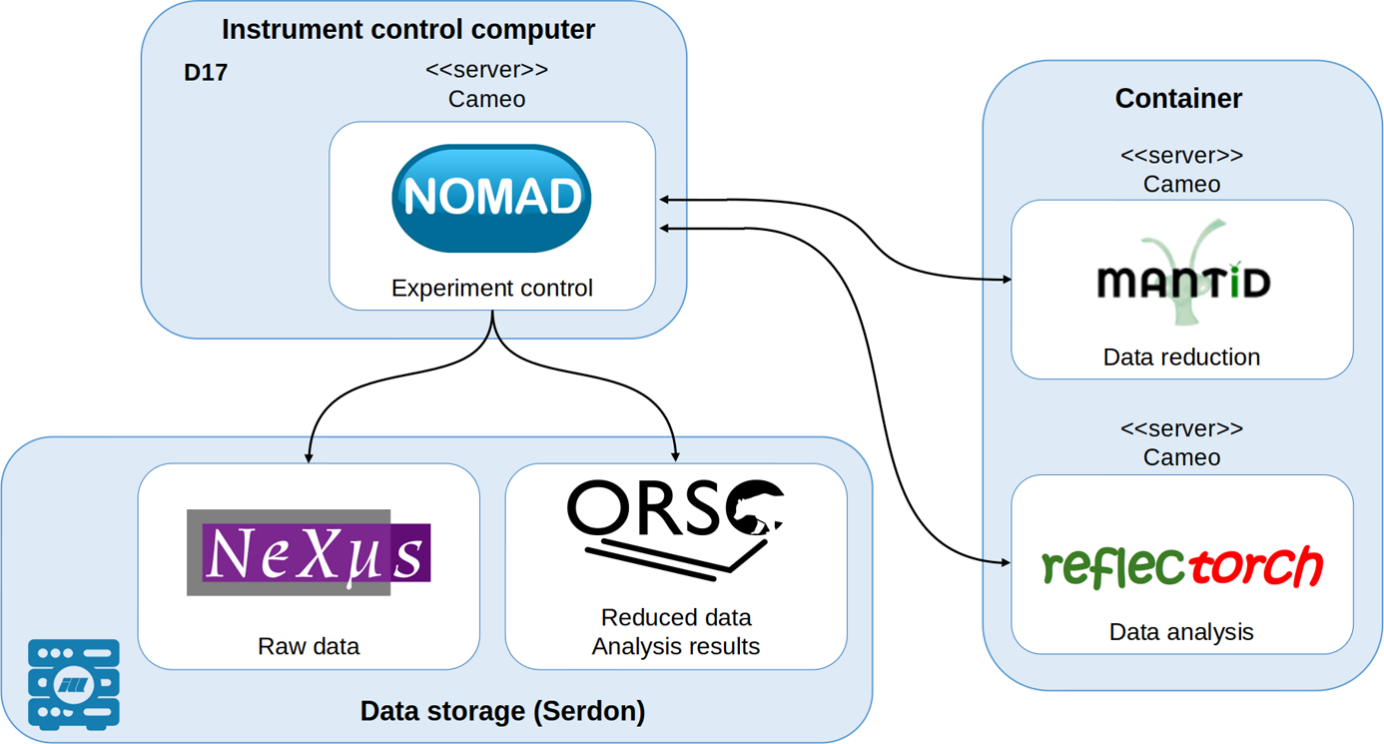
Illustration of the updated workflow. Both *Mantid* and *reflectorch* have a feedback connection to *NOMAD*. The measured curves are saved on Serdon by *NOMAD*.

This section outlines the software components and the workflow established to facilitate real-time reflectometry data analysis. Fig. 1 ▸ shows the automated workflow using the IT infrastructure of the ILL; Fig. S1 shows a preliminary version of this workflow. While the implementations were tested on the D17 instrument (Saerbeck *et al.*, 2018 ▸) at the ILL, these approaches can potentially be extended to other instruments and facilities as well.

### 2.1. CAMEO

*CAMEO* (https://code.ill.fr/cameo/cameo.wiki.git) is a remote application manager that coordinates the operation of multiple software components. It manages the communication between applications and controls their execution. *CAMEO* enables the exchange of information between different apps using JSON-formatted messages or binary data. *CAMEO* is the software of choice at the ILL and we used it to establish our workflow. Of course, other software with similar functionality could, in principle, be considered as well. In the workflow illustrated in Fig. 1 ▸, the synchronization of the individual applications is handled by *CAMEO*. The application server listens for messages and returns an answer when the initiated process is completed.

### 2.2. Experiment control (*NOMAD*)

*NOMAD* (Mutti *et al.*, 2011 ▸) is the software responsible for the control of the instruments at the ILL. It runs on the instrument control computer and is responsible for general experiment orchestration and data acquisition. The messaging to other software components is implemented via the remote application manager *CAMEO*. It is possible to trigger any action, *e.g.* data reduction and analysis, from *NOMAD*, as well as receive feedback from the data analysis software and take actions accordingly, as described in Section 2.6 below. On D17, *NOMAD* saves the measured raw data and experimental metadata in NeXus format (Jemian *et al.*, 2024 ▸) on the Serdon data storage server, which can be accessed from other machines in the ILL intranet. By default, it saves the data every 30 s during a measurement, but this frequency can be adjusted.

### 2.3. Data analysis environment (*VISA*)

*Virtual Infrastructure for Scientific Analysis* (*VISA*) is a virtual machine manager used at the ILL and ESRF. Virtual­ization enables the creation of isolated computing environments sharing the same hardware (Smith & Nair, 2005 ▸). This allows flexibility and enhanced security through separate instances. Each instance can have a different configuration of virtual CPUs (optionally GPUs), as well as configurable memory. At the ILL, it enables access to experimental data stored on the Serdon data storage server and provides computational resources for data analysis for general users of the facility (https://visa.readthedocs.io/). Additionally, it provides an opportunity for remote control of experiments via *NOMAD Remote*.

### 2.4. Data reduction (*Mantid*)

*Manipulation and Analysis Toolkit for Instrument Data* (*Mantid*) is a free open source software project for the analysis of neutron scattering and muon spectroscopy data supported by many large-scale facilities worldwide (Arnold *et al.*, 2014 ▸). We used the internal version supported by the ILL for the reduction of reflectometry data, which is based on the algorithm described by Gutfreund *et al.* (2018 ▸). This version is available at *VISA* instances for users by default. Data reduction in the case of NR experiments includes conversion from wavelength and angular space to *q* space, normalization to the incoming neutron flux, and background scattering subtraction.

### 2.5. Data analysis

To enable fast analysis of the recorded experimental data we make use of the Python library *reflectorch* (Munteanu *et al.*, 2025 ▸) which facilitates the training and deployment of ML models for the analysis of NR and XRR data. A comprehensive description of the ML approach is available in the report by Munteanu *et al.* (2024 ▸). Here we give a concise overview of the technical functionalities of the package and highlight updates and differences relevant to NR compared with the earlier publication. The trained models are designed to infer thin-film parameters from reflectometry data by learning the inverse mapping from reflectivity curves to physical film properties.

The ML models are implemented as neural networks that take as input the reflectivity curve (reflectivity *R* and momentum transfer *q*) and a set of upper and lower bounds (prior bounds) for the physical parameters of interest, representing the prior knowledge of the scientists. The thin-film parameters output by the network can be further polished by performing a least-squares fit to the experimental data, with the initial network predictions serving as a starting point. This polishing step is performed using the *SciPy* package (Virtanen *et al.*, 2020 ▸), specifically the *Trust Region Reflective* algorithm (Coleman & Li, 1996 ▸). If the uncertainty of the reflectivity Δ*R* is provided as an input to this fit, the weighted sum of squares is minimized (*i.e.* χ^2^). Additionally, the prior bounds serve as a constraint to the solution space during this step. The co­variance matrix and residuals obtained from the fitting algorithm are used to calculate the uncertainties of the fitting parameters reported below. The neural networks trained for this application consist of an integral convolutional embedding network that encodes *q*-dependent features of the reflectivity curves into a latent space. This embedding is then processed by a residual multilayer perceptron.

The employed parameterization for simulating the reflectivity curves includes the sample parameters (thicknesses, roughnesses and SLDs) and two further parameters, namely, the scaling factor for the intensity of the curve and the constant background. While the overall neural network architecture is similar, a key difference between the networks used for XRR and NR analysis is the application of resolution smearing, Δ*q*/*q*, to the reflectivity curves during training, where Δ*q* denotes the uncertainty in *q*. The quantity also serves as an additional input to the neural network. During inference for data with pointwise resolution smearing, this network input is computed as the average over the resolution array, but the full resolution array is used during the polishing step. Since points with large intensity errors in the experimental data can negatively influence the neural network prediction, a preprocessing step is applied where the points that have a ratio of the intensity error to intensity value above a certain threshold (here 0.3) are filtered out from the input to the neural network. However, the polishing step still uses the full data set.

Pretrained models for *reflectorch* (*i.e.* neural network weights and their corresponding configuration files) are hosted in a Hugging Face repository (https://huggingface.co/reflectorch-ILL) which allows them to be automatically downloaded and deployed on new hardware. For establishing our data analysis workflow here, we use models parameterized for samples with one and two layers. The parameter ranges used for training the two networks can be found in Table S1.

The prior-amortized approach proposed by Munteanu *et al.* (2024 ▸) and efficient embedding networks (Starostin *et al.*, 2025 ▸) provide the best performance. For multimodal distributions and Bayesian analysis, we plan to incorporate the recent approach using simulation-based inference (Starostin *et al.*, 2025 ▸; Deistler *et al.*, 2025 ▸) into *reflectorch*. We note that there are other ML efforts dedicated to reflectometry and other scattering techniques, such as those reported by Doucet *et al.* (2021 ▸), Mironov *et al.* (2021 ▸) and Hoogerheide & Heinrich (2024 ▸), and also from within our group (Greco *et al.*, 2019 ▸; Greco *et al.*, 2021 ▸; Greco *et al.*, 2022 ▸; Hinderhofer *et al.*, 2023 ▸; Völter *et al.*, 2025 ▸).

### 2.6. Integration for on-the-fly analysis

The implemented workflow, consisting of the elements described above, is illustrated in Fig. 1 ▸. *NOMAD* is deployed as a *CAMEO* application server. When the *NOMAD* server is started, it launches both *Mantid* and *reflectorch*, which are run within a containerized setup, each with its own separate *CAMEO* application server. Messaging between the servers via *CAMEO* ensures seamless and stable communication among all components. The selected *reflectorch* ML models are loaded into memory once when the corresponding server is initiated. During the measurements, *NOMAD* collects the raw detector data and automatically sends them for reduction to *Mantid* at the frequency specified by the user. Frequencies between 10 and 30 s were tested. *Mantid*, when it receives the raw data from *NOMAD*, reduces them to a set of values (*q*, Δ*q*, *R* and Δ*R*) and sends these reduced reflectivity data back to *NOMAD*. *NOMAD* receives the reduced data, saves them in ASCII (.mft) format and triggers the *reflectorch* server to start the analysis. Each time the analysis is initiated, *NOMAD* sends a message with the reduced experimental data, which ML model to use, the current prior bounds and additional metadata. Therefore, it is possible to switch between different *reflectorch* models during an ongoing experiment and to update the prior bounds continuously. Once the analysis of the reduced experimental data is completed, *reflectorch* prepares the results (predicted and polished parameters, and reflectivity curves simulated with these values) for export in ORSO format (Glavic *et al.*, 2024 ▸). The resulting file is returned to *NOMAD* in ASCII form and ultimately stored on Serdon with the .ort file extension. An example of the produced ORSO files is provided in the supporting information. The predicted parameters, the reflectivity curve simulated from these values and the curve computed using the polished predictions are also sent back to *NOMAD*. To provide immediate visual feedback, the live plot feature of *NOMAD* displays the predicted and polished curves together with the experimental data. The predicted parameters are simultaneously shown in the *NOMAD* control panel, allowing users to monitor the results in real time. This is especially useful for *in situ* experiments, where the studied sample undergoes dynamic transformations. To demonstrate the ability of the described workflow to close the loop, an if condition was added into the experimental workflow, and when the specified condition was met this was recorded in the log files. Thus, this setup provides a pathway toward real-time adaptive control of the experiment.

## 3. Experiment

We tested a preliminary version of the on-the-fly analysis workflow during an NR experiment on the D17 instrument at the ILL. A detailed description of this version of the pipeline can be found in Section S1. The different software components were run on a private *VISA* instance (Section 2.3). Additionally, *refnx* (Nelson & Prescott, 2019 ▸) was incorporated into this workflow as a benchmark for our ML-based approach.

The experiment was dedicated to studying the mixing behaviour in bilayer thin films relevant for applications in organic photovoltaics. Two polymer/small-molecule samples were studied. The first was a polymer/non-fullerene bilayer on a silicon substrate [with a 15 Å native oxide layer (Higgins *et al.*, 2023 ▸; Higgins *et al.*, 2024 ▸)], initially consisting of a deuterated polystyrene (d-PS) top layer (weight-average mol­ecular weight *M*_w_ = 5.2 kg mol^−1^) on top of a 5,5′-[(9,9-dioctyl-9*H*-fluorene-2,7-diyl)bis(2,1,3-benzothiadiazole-7,4-diylmethyl­idyne)]bis[3-ethyl-2-thioxo-4-thiazolidinone] (FBR) (Holliday *et al.*, 2015 ▸) bottom layer. The second sample initially consisted of a top layer that was a blend of hydrogenated polystyrene (h-PS) (*M*_w_ = 295 kg mol^−1^) and the fullerene bis-adduct phenyl-C61-butyric acid methyl ester (bis-PCBM) on top of a bis-PCBM bottom layer. The blend layer was 8 wt% bis-PCBM and 92 wt% h-PS. The FBR, bis-PCBM and h-PS–bis-PCBM-blend solutions were prepared in chlorobenzene. The d-PS was dissolved in toluene. The bottom layers were then formed by spin-coating either FBR solution or bis-PCBM solution onto the polished faces of silicon substrates. The top layers were prepared by spin-coating solutions (of either d-PS or the h-PS–bis-PCBM-blend) onto sheets of freshly cleaved mica and then floating the layers onto the surface of a bath of deionized water. This floating layer was then deposited onto the silicon/bottom layer to make a bilayer sample [see the supporting information reported by Higgins *et al.* (2024 ▸)]. Additional information on the sample preparation is given in Section S5.

### 3.1. *In situ* NR

The samples were bolted onto the surface of a heater block within a vacuum chamber on the reflectometer D17 at the ILL. The setup was the same as that described by Higgins *et al.* (2023 ▸) and Higgins *et al.* (2024 ▸), except for two modifications: firstly a thin layer of thermal paste was placed between the heating block and the underside of the sample, and secondly a PT100 temperature sensor was placed in contact with the underside of the sample to record the temperature during *in situ* thermal annealing [these are the temperatures given throughout the paper, *e.g.* in Fig. 3(*a*)].

The chamber was pumped down to a pressure of around 10^−5^ mbar using a turbo pump. The sample was then aligned and the *in situ* thermal annealing protocols started. NR measurement consisted of acquisition using a white beam of neutrons in time-of-flight mode at incidence angles of 0.8° and 3°. By using relatively relaxed resolutions Δ*q*/*q* of around 2% at *q* = 0.008 Å^−1^ and of around 8% at *q* = 0.15 Å^−1^, we were able to collect a full NR curve (up to *q* ≃ 0.2 Å^−1^) with good statistics using a total of 10 min of acquisition time (2 and 8 min acquisition times for incidence angles of 0.8° and 3°, respectively). The heater set-point temperature was then changed in a stepwise fashion. Full NR measurements were taken after waiting a sufficient time for the temperature to stabilize (Higgins *et al.*, 2023 ▸) (*e.g.* this was typically 6 min for an approximately 10 K temperature increase and 10 min for an approximately 10 K temperature decrease for the fullerene bilayer). During the temperature stabilization process, NR measurements were taken at an incidence angle of 0.8° only.

### 3.2. Analysis details

**Figure 2:**
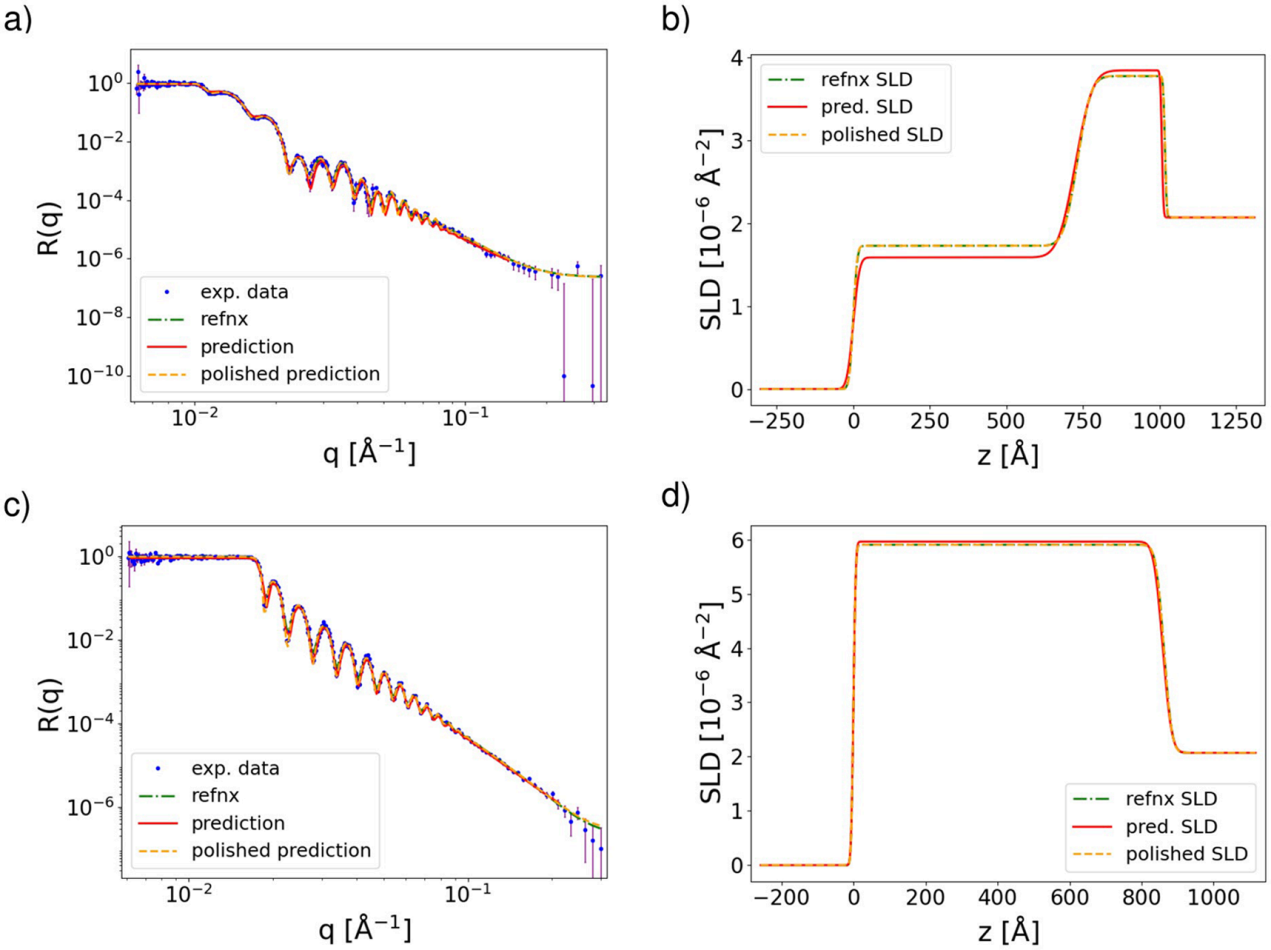
Fitting results for (*a*) and (*b*) the h-PS/bis-PCBM and (*c*) and (*d*) the d-PS/FBR samples. These are typical measurements and fits during an *in situ* annealing process. (*a*) and (*c*) Experimental data and corresponding fits. Blue points show the measured reflectivity data, the red and orange curves are calculated for the parameters predicted by *reflectorch* (red) and subsequently polished by least-squares fitting (orange), and the green curve is calculated for the parameters extracted by *refnx*. (*b*) and (*d*) SLD profiles corresponding to the parameters extracted by *reflectorch* and *refnx*. Panels (*a*) and (*c*) are shown on an *R*(*q*) *q*^4^ scale in Figs. S2 and S4, respectively.

Fig. 2 ▸ shows examples of the analysis of the NR curves measured from the samples described above. The experimental curve of the (as fabricated) h-PS/bis-PCBM sample measured at 437.2 K shown in Fig. 2 ▸(*a*) contains Kiessig fringes with different periods, indicating a multilayer structure. On the other hand, the curve measured for the d-PS/FBR sample at 377.7 K shows Kiessig fringes consistent with a single period without any additional modulations, suggesting that a one-layer model is appropriate. Consequently, we used different *reflectorch* models to analyse these samples. Due to the low contrast between the FBR layer and the silicon substrate (in comparison with the SLD of the d-PS top layer), the bottom (FBR) layer thickness is not readily discernible from NR measurements of the d-PS/FBR bilayer. This similarity in the SLDs makes it almost impossible to determine the parameters of the FBR layer. Atomic force microscopy and previous NR measurements on single FBR layers spin-coated from FBR solutions of similar concentration at similar spin speeds gave FBR layer thicknesses of 53 and 58 nm, respectively.

The substrate SLD ρ_0_ was fixed at 2.07 × 10^−6^ Å^−2^ for both reflectivity curves, with air as the fronting medium. The data collected for the h-PS/bis-PCBM sample were analysed using an ML model trained for the analysis of two layer films. There are seven free parameters [thickness *h*_2_, roughness σ_2_ and SLD ρ_2_ of the top (h-PS) layer, thickness *h*_1_, roughness σ_1_ and SLD ρ_1_ of the bottom (bis-PCBM) layer, and substrate roughness σ_0_]. The data for the d-PS/FBR sample were analysed using a one-layer ML model with four adjustable parameters [thickness *h*, roughness σ and SLD ρ of the top (d-PS) layer, and substrate roughness σ_0_]. In both cases, the scaling factor and background level were additional free parameters. The native silicon oxide layer, the inclusion of which could marginally improve the quality of the fits, was not included in the model as it has little impact on the fitted parameters for the layers above and no impact on the decision-making process during the experiment.

All free parameters were extracted using both *reflectorch* and *refnx*. The *reflectorch* analysis yields two sets of parameters, one corresponding to the raw neural network predictions and another obtained by polishing the initial prediction through a least-squares fit as described in Section 2.5. The analysis in *refnx* yields a single set of parameters obtained by a differential evolution fit. For both packages, we used the same prior bounds for the adjustable parameters. The goal here is not to compare the two analysis methods but just to demonstrate the fitting capability of *reflectorch* relative to an established analysis program. Details of the prior bounds are given in the supporting information, Sections S3 and S4.

The curves corresponding to the parameters extracted by both analysis packages (show in Tables 1 ▸ and 2 ▸) are shown in Figs. 2 ▸(*a*) and 2 ▸(*c*), along with the measured curves. As can be seen from the figures, all simulated curves overlap with the experimental data with a high degree of accuracy. Indeed, the parameters extracted by *reflectorch* after the polishing step and fitted with *refnx* are in good agreement within the estimated error margins. The raw predicted values from *reflectorch* deviate slightly from those values, but are quite close to the polished values and provide a good initial guess for the subsequent polishing step. This deviation can also be seen in the corresponding SLD profiles shown in Figs. 2 ▸(*b*) and 2 ▸(*d*). We note that a Markov chain Monte Carlo fit might provide still more reliable values of the thin-film parameters and their uncertainties, but this would be beyond the scope of this study.

### 3.3. *In situ* experiment

The data analysis routine described above was employed for the analysis of the results of *in situ* measurements performed on these samples. Here we demonstrate the results for the h-PS/bis-PCBM sample collected during temperature variation in the range 353–467 K, which took about 404 min and resulted in 100 measured NR curves. Some of the curves were measured only at the first incidence angle for higher temporal resolution during rapid temperature changes.

**Figure 3:**
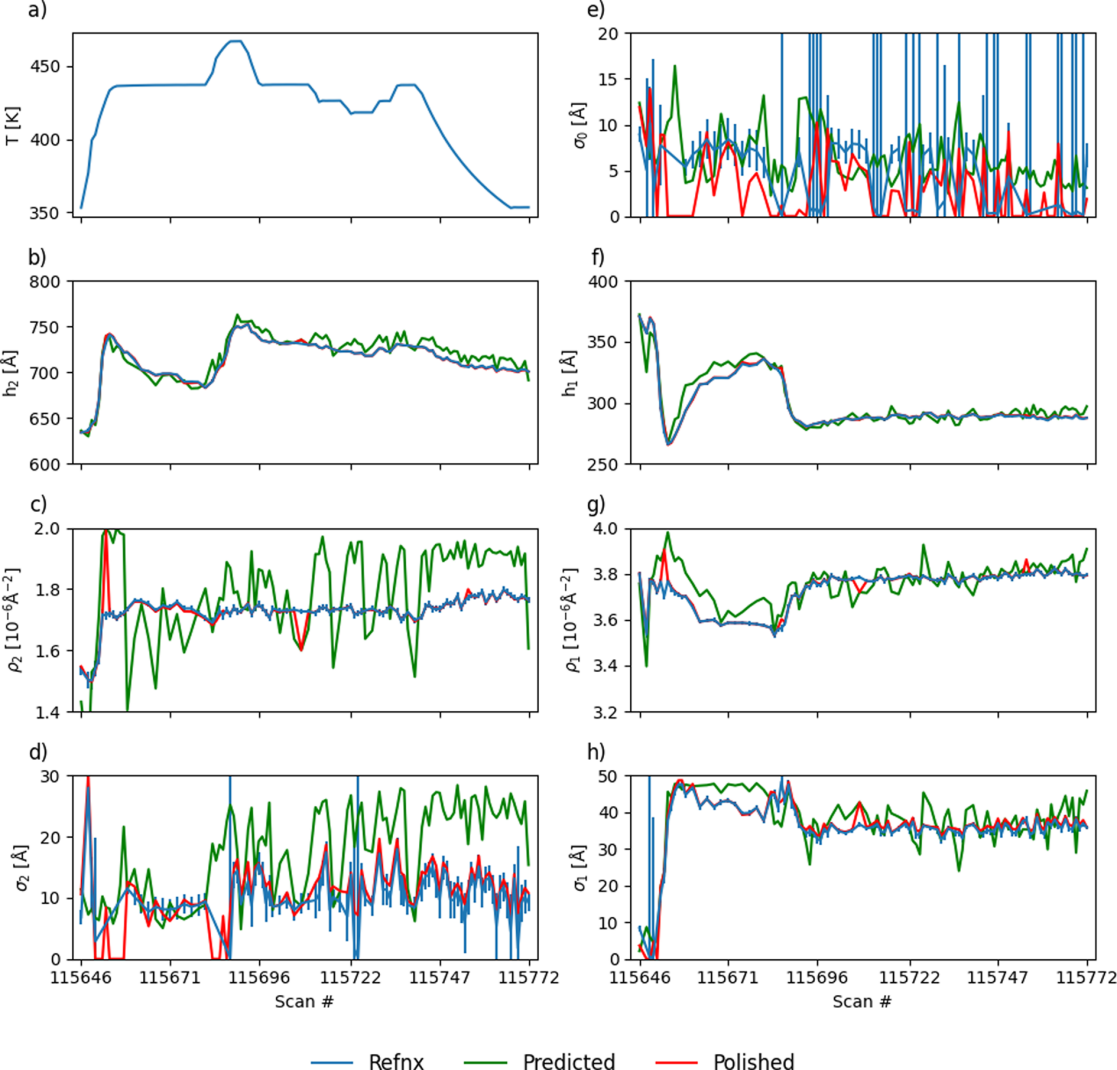
Variation in sample parameters during *in situ* annealing at different temperatures for the h-PS/bis-PCBM sample: (*a*) temperature, (*b*) top layer thickness *h*_2_, (*c*) top layer SLD ρ_2_, (*d*) top layer roughness σ_2_, (*e*) substrate roughness σ_0_, (*f*) bottom layer thickness *h*_1_, (*g*) bottom layer SLD ρ_1_ and (*h*) bottom layer roughness σ_1_. The blue curves show the parameters extracted by *refnx*, the green curves show the raw predicted parameters provided by *reflectorch* and the results after the polishing step are in red. We have omitted the error bars of the *refnx* fit where the value approached zero (lower boundary) as those parameter uncertainties are not meaningful. For clarity, we also omitted the uncertainties of the parameters obtained from the polished *reflectorch* predictions.

Fig. 3 ▸ shows how the parameters characterizing the sample vary with changing annealing temperature. The analysis results were obtained using two-layer *reflectorch* and *refnx* models. In general, there is no guarantee that a two-layer model provides the best fit for the h-PS/bis-PCBM sample at all temperatures. However, a bilayer model was successfully used in previous studies (Higgins *et al.*, 2023 ▸; Higgins *et al.*, 2024 ▸) and was therefore adopted for the present analysis.

For both layer thicknesses, the two employed analysis methods return parameters that align closely. While the predictions for the SLDs deviate from the *refnx* plot, the polished predictions match the *refnx* fits visually very well for the majority of points. The fits for the roughness of the substrate have large error bars. This is also true for the roughness of the layers, although the results of the analysis methods do not deviate as much. The analysis of the variation in sample parameters during the *in situ* d-PS/FBR experiment is shown in Fig. S3.

Table 3 ▸ shows the average computation time for the analysis of a single reflectivity curve with *reflectorch* and *refnx*. The 100 curves measured during the *in situ* experiment were analysed. Measurements were taken at the first incidence angle, with some curves additionally measured at the second angle. The resulting curves were then combined. For two of these curves, the least-squares fit implemented in *SciPy* did not return any results for the polished parameters. In both cases, the fitting process reached the maximum number of iterations, which caused a significant delay. Consequently, the time required to analyse these two curves was excluded from the average calculation time for the results involving polishing with the CPU. Appropriate error handling is implemented to ensure that the workflow proceeds uninterrupted even if an error occurs during analysis of the experimental data. This means that a consequence of the minimum not being found is that the workflow returns nonideal results, which does not have a big impact as the frequency of this happening is low compared with the high frequency with which the workflow is triggered. The number of iterations for the least-squares fit can be adjusted.

The results in Table 3 ▸ demonstrate a clear improvement in computational efficiency, with *reflectorch* taking a shorter average analysis time across all the hardware configurations tested than the conventional tool *refnx*. While the advantage in speed is not a necessity for the system presented, other experiments where the measurement time can be much shorter, down to 100 ms per curve (Saerbeck *et al.*, 2018 ▸), will benefit. Fast ML-based data analysis brings added value to the experiment (Aoki *et al.*, 2021 ▸), since this allows evaluation of the data quality *during* the scan, which enables the natural next step, *i.e.* closed-loop experiments (Pithan *et al.*, 2023 ▸).

### 3.4. Towards closed-loop experiments

**Figure 4:**
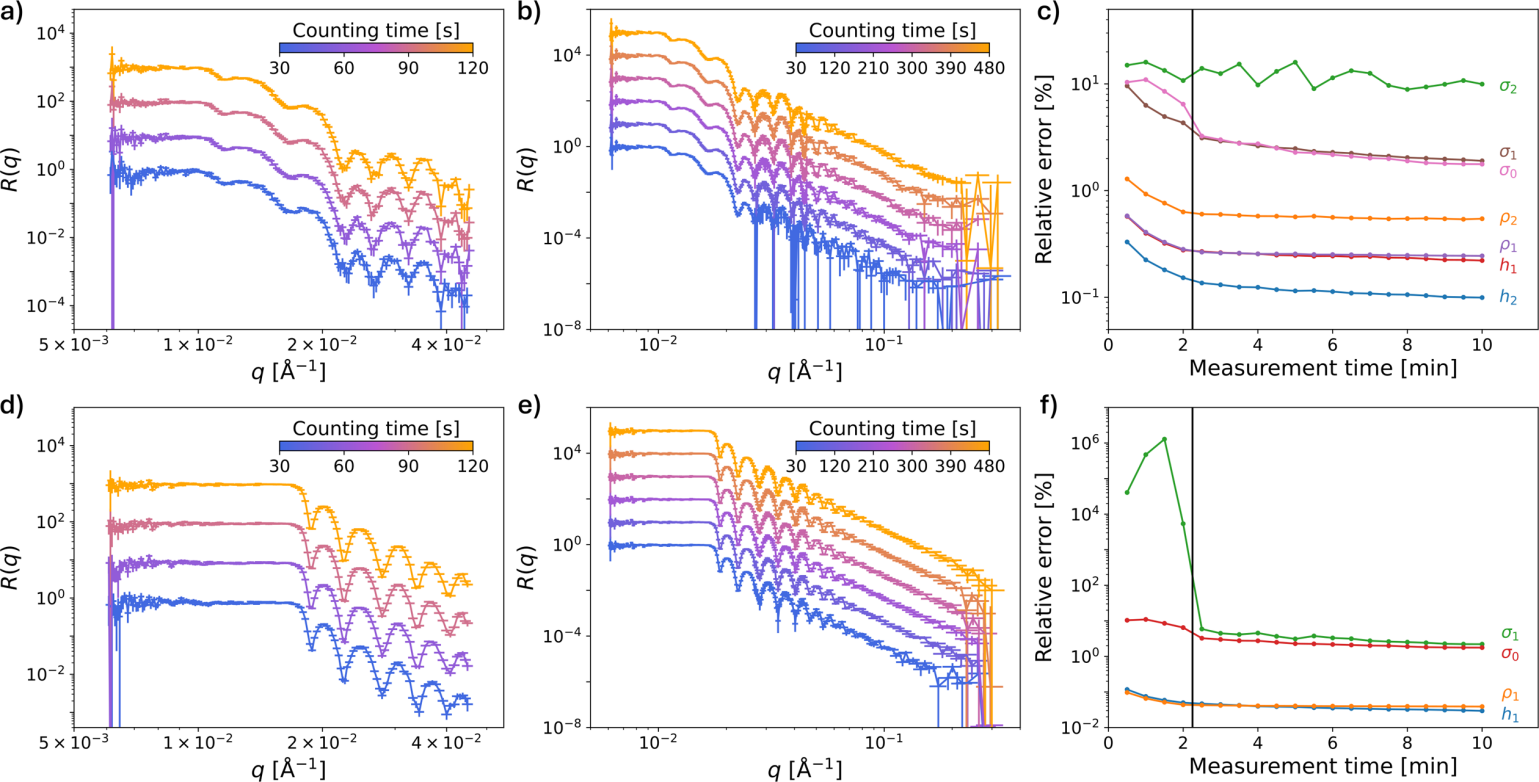
Evolution of the errors of the extracted parameters with measurement time for (*a*)–(*c*) the h-PS/bis-PCBM and (*d*)–(*f*) the d-PS/FBR samples. (*a*) and (*d*) NR curves measured with different counting times at the first incidence angle (0.8°). (*b*) and (*e*) NR curves measured at the second angle (3.0°) with different counting times, concatenated with the first section of the curve measured at the first angle. The reflectivity curves measured at a higher counting time are shifted for visibility. (*c*) and (*f*) The relative error of the parameters obtained by fitting the curves in panels (*a*), (*b*), (*d*) and (*e*) as a function of measurement time. The vertical black lines indicate the point at which the incidence angle was changed.

The implemented on-the-fly data reduction and analysis allowed us to track potential ambiguity in the extracted parameters with counting time during the measurements. Fig. 4 ▸ illustrates the results of this analysis.

Obviously, the uncertainties in the measured intensities decrease as the counting time increases, allowing extraction of the parameters with higher precision. However, some of the parameters (namely, the roughness values for different interfaces) do not follow this trend and remain high (very high in some cases) during the measurements taken at the first incidence angle. This indicates the necessity of performing the measurements at the second incidence angle, and analysing the reflectivity profile resulting from concatenating the first and second parts of the reflectivity curve, measured at the first and second angles, respectively, to extract these parameters with reasonable accuracy. Analysing these parameters at high temporal resolution (*i.e.* high analysis frequency) allows the measurement to be stopped once a predefined error threshold of the parameters of interest is reached or when the error no longer decreases significantly. For instance, in the h-PS/bis-PCBM sample, the error value of σ_2_ did not decrease in a significant way. For this experiment, this parameter is of little interest. Therefore, the second angle measurement could potentially have been stopped earlier, once the error values of the other parameters no longer decreased substantially. The frequency at which the data were reduced and analysed was successfully tested at 10 s, though higher frequencies might be feasible.

## 4. Conclusions and outlook

We have demonstrated a pipeline that analyses experimental data on the fly using *reflectorch*. To validate the results, we used those obtained by the conventional data analysis software *refnx*. The raw predicted parameters computed using *reflectorch* provide a good starting point for a subsequent polishing step. When analysing individual reflectivity curves, the polished parameters computed with *reflectorch* overlap with the fitted parameters within their respective uncertainty limits.

The ML-based analysis is very fast and computationally inexpensive. Using GPU acceleration, predicting the parameters with *reflectorch* is more than 100 times faster than fitting the parameters with a conventional analysis program, and up to ten times faster on a CPU when including the polishing step.

The processing efficiency of the ML-based software facilitates real-time analysis during experiments. This is particularly valuable during *in situ* experiments, where the evolution of the sample during deposition or the response of the sample to changing conditions is observed. The analysis results transmitted to *NOMAD* support data-driven decisions for the subsequent steps of the experiment.

The proposed workflow can reliably trigger the automated analysis pipeline at intervals of 10 s, thus enabling detailed tracking of the evolution of the analysed sample parameters and their corresponding relative errors over the exposure time.

Together, the real-time analysis, the fine-grained monitoring of relative parameter uncertainties, and the feedback con­nec­tion between *reflectorch* and *NOMAD* represent a concrete step toward closed-loop experimental workflows. This could, for example, enable automatic adjustment of the incidence angle once a target relative error is reached, or experimental parameters such as temperature could be modified when specific layer properties are detected. The analysis results obtained with *reflectorch* during *in situ* experiments could be further enhanced by dynamically adapting the prior bounds according to the outcomes of previous analyses. By iteratively updating these bounds, additional prior knowledge can be incorporated into the workflow, potentially enhancing the accuracy of the extracted parameters.

## Supplementary Material

Additional figures and tables. DOI: 10.1107/S1600576726002657/uz5030sup1.pdf

Example ORSO file. DOI: 10.1107/S1600576726002657/uz5030sup2.txt

## Figures and Tables

**Table 1:** The parameters extracted with *reflectorch* and *refnx* for the h-PS/bis-PCBM sample

	*reflectorch*		*refnx*
Parameter	Predicted	Polished	Fit
*h*_2_ (Å)	724.8	731.6 ± 0.7	731.3 ± 0.7
*h*_1_ (Å)	282.4	286.1 ± 0.6	285.9 ± 0.6
σ_2_ (Å)	16.1	10.0 ± 1.2	10.6 ± 1.0
σ_1_ (Å)	44.4	35.1 ± 0.7	35.7 ± 0.7
σ_0_ (Å)	4.2	4.8 ± 1.3	4.6 ± 1.2
ρ_2_ (10^−6^ Å^−2^)	1.6	1.73 ± 0.01	1.73 ± 0.01
ρ_1_ (10^−6^ Å^−2^)	3.8	3.78 ± 0.01	3.77 ± 0.01
*r*_scale_	0.9	0.94 ± 0.01	0.94 ± 0.01
log_10_ background	−6.9	−6.6 ± 0.2	−6.6 ± 0.1

**Table 2:** The parameters extracted with *reflectorch* and *refnx* for the d-PS/FBR sample

	*reflectorch*		*refnx*
Parameter	Predicted	Polished	Fit
*h* (Å)	858.5	861.7 ± 0.3	861.8 ± 0.3
σ (Å)	5.1	5.0 ± 0.1	4.9 ± 0.1
σ_0_ (Å)	19.6	17.7 ± 0.4	17.9 ± 0.3
ρ (10^−6^ Å^−2^)	6.0	5.903 ± 0.002	5.903 ± 0.002
*r*_scale_	0.9	0.968 ± 0.003	0.968 ± 0.002
log_10_ background	−6.4	−6.6 ± 0.2	−6.6 ± 0.1

**Table 3:** Evaluation of computation time for *reflectorch* and *refnx* analyses The values show the time taken to fit a single curve averaged over the total number of considered curves.

	*reflectorch*			
	CPU		GPU	*refnx*
	With polishing (s)	Prediction (s)	Prediction (s)	Parameters (s)
CPU *VISA*; 16 GB RAM, 4 VCPUs	0.9 ± 0.4	0.2 ± 0.1	–	7.9 ± 1.9
GPU *VISA*; 32 GB RAM, 16 VCPUs	0.4 ± 0.1	0.1 ± 0.1	0.05 ± 0.10	7.2 ± 1.5
Workstation; 32 GB RAM, 6 CPUs	0.5 ± 0.4	0.19 ± 0.01	0.02 ± 0.03	6.3 ± 2.6

## Data Availability

Data collected during the D17 beamtime can be accessed at https://doi.org/10.5291/ILL-DATA.9-11-2246.
