# Supplementary material for: Towards machine-learning-based on-the-fly analysis of neutron reflectometry
Source: J Appl Crystallogr. 2026 May 14;59(Pt 3):765–73. doi: 10.1107/S1600576726002657 (PMC13224789; doi:10.1107/S1600576726002657)
Supplement: Supplementary file 1 [file j-59-00765-sup1.pdf]

Supplementary information
Towards machine-learning-based on-the-fly analysis of neutron reflectometry

Anne Rentzsch [a], Valentin Munteanu [a], Oliver Anyanor [b], Shreya Shah [a], Philipp Gutfreund [c], Rémi Perenon [c], Anthony Higgins [b], Vladimir Starostin [d], Alexander Hinderhofer [d], Dmitry Lapkin [d], and Frank Schreiber [a]

[a]Institut für Angewandte Physik, Universität Tübingen, 72076 Tübingen, Germany
[b]School of Engineering and Applied Science, Swansea University, Swansea SA1 8EN, Wales, United Kingdom
[c]Institut Laue-Langevin, 38000 Grenoble, France
[d]Cluster of Excellence "Machine learning - new perspectives for science", Universität Tübingen, Maria-von-Linden-Straße 6, 72076 Tübingen, Germany

S1 First version of the established workflow

In our first tests, we triggered the data reduction and analysis in a user-created VISA instance every time the data on the server was updated. All data reduction and analysis were done in Python. We tested different configurations of the VISA instances as discussed below (Section 3.3) an illustration of this first version of the workflow is on Figure S1. We employed a file watcher to track changes in the raw data files every 1 s. When a raw data file was modified, it triggered the data reduction by `Mantid`, resulting a standard four column reflectivity data file with the momentum transfer q, reflectivity R, ΔR and Δq. The reduced data are passed further to `reflectorch` (Munteanu *et al.*, 2024) or `refnx` (Nelson & Prescott, 2019) packages for analysis, where layer parameters (SLD, thickness, roughness) are extracted. After the analysis, the reduced data and the extracted layer parameters are saved in the ORSO format (Glavic *et al.*, 2024) to Serdon.

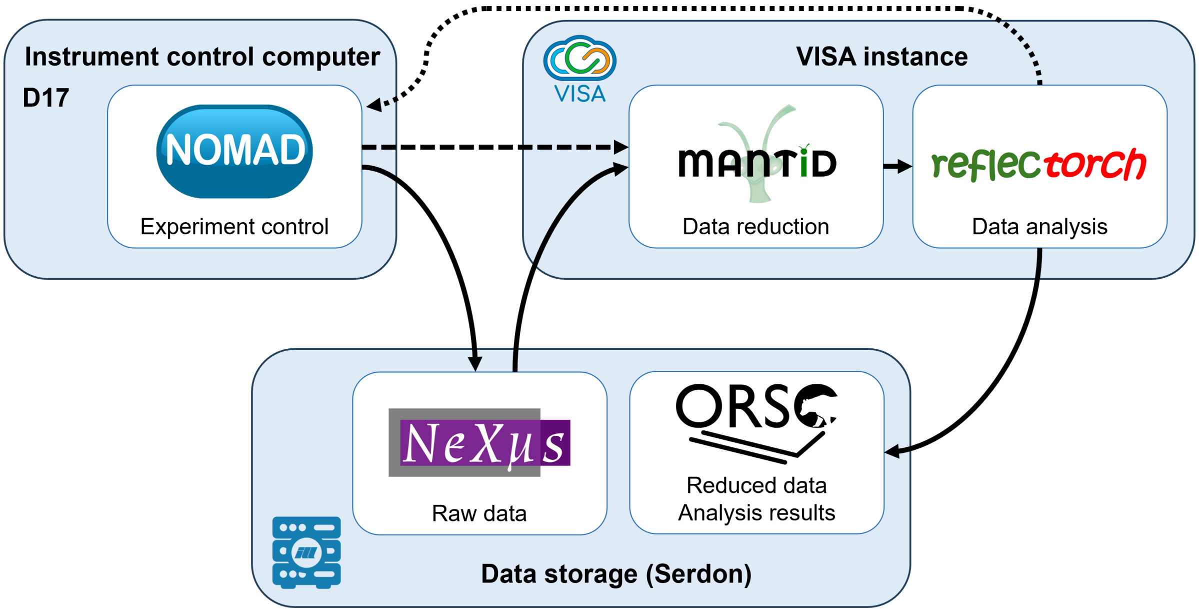

Figure S1: Illustration of the software components involved in the on-the-fly data analysis pipeline, as implemented at the D17 instrument at the ILL. Solid lines indicate the implemented parts of the workflow. The dashed arrow shows the intended triggering of the data reduction and analysis directly from the instrument control system NOMAD. The dotted line connecting `reflectorch` to NOMAD shows the potential feedback loop.

S2 Training ranges of the `reflectorch` networks

Table S1: Training ranges for the one and two-layer `reflectorch` model.

	Parameter range	Prior bound range
h [Å]	[1.0, 1500.0]	[1.0×10^{-2}, 1500.0]
σ [Å]	[0.0, 60.0]	[1.0×10^{-2}, 60.0]
ρ [10^{-6} Å^{-2}]	[-8.0, 16.0]	[1.0×10^{-2}, 5.0]
r_{scale}	[0.9, 1.1]	[1.0×10^{-3}, 0.2]
$\log_{10}$ background	[-10.0, -4.0]	[1.0×10^{-2}, 6.0]

S3 Two-layer example

Table S2: Prior bounds for the *in situ* example, Figure 3, used for both analysis methods.

Parameter	Lower bound	Upper bound
h_2 [Å]	400.0	800.0
h_1 [Å]	100.0	500.0
σ_2 [Å]	0.01	30.0
σ_1 [Å]	0.01	50.0
σ_0 [Å]	0.01	20.0
ρ_2 [10^{-6} Å^{-2}]	1.0	2.0
ρ_1 [10^{-6} Å^{-2}]	2.0	5.0
ρ_0 [10^{-6} Å^{-2}]	2.07	2.07
r_{scale}	0.91	1.1
$\log_{10}$ background	-9.0	-4.3

Table S3: Prior bounds for the two-layer `reflectorch` model analysis and the `refnx` fit, the results are visualized in Figure 2a,b.

Parameter	Lower bound	Upper bound
h_2 [Å]	700.0	800.0
h_1 [Å]	250.0	300.0
σ_2 [Å]	10.0	30.0
σ_1 [Å]	20.0	50.0
σ_0 [Å] [Å]	0.01	50.0
ρ_2 [10^{-6} Å^{-2}]	1.4	2.0
ρ_1 [10^{-6} Å^{-2}]	3.5	4.0
ρ_0 [10^{-6} Å^{-2}]	2.07	2.07
r_{scale}	0.91	1.1
$\log_{10}$ background	-7.0	-4.3

Table S4: Parameters defining the layers of the two-layer `refnx` model parameters. Addition to Table S3.

Parameter	Value
h_2 [Å]	732
h_1 [Å]	285
σ_2r [Å]	20
σ_1 [Å]	35
σ_0 [Å]	4
ρ_2 [10^{-6} Å^{-2}]	1.72
ρ_1 [10^{-6} Å^{-2}]	3.77
r_{scale}	1.0
$\log_{10}$ background	-6

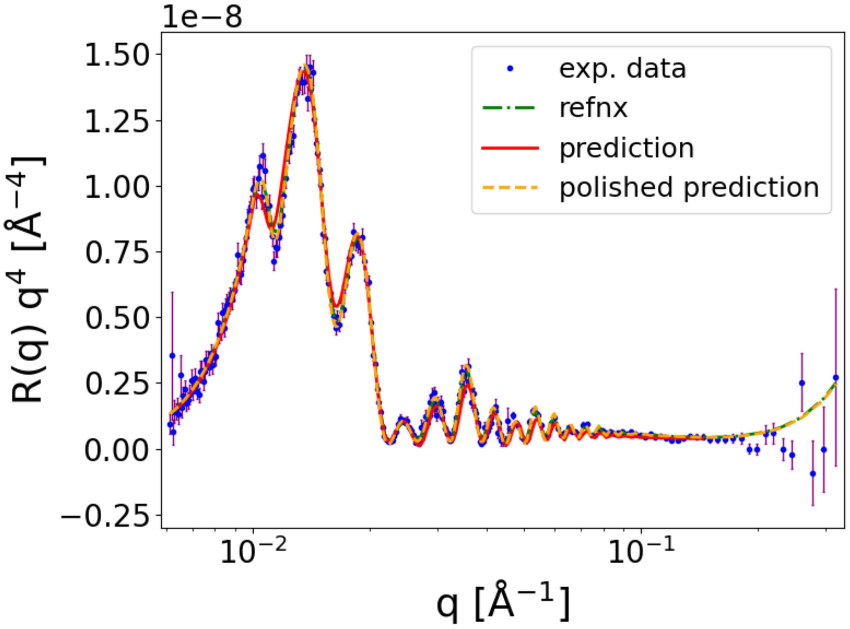

Figure S2: Fitting results for the h-PS/bis-PCBM sample. Addition to Figure 2(a).

S4 One-layer example

Table S5: Prior bounds for the one-layer `reflectorch` model analysis and the `refnx` fit, the results are visualized in Figure 2c,d.

Parameter	Lower bound	Upper bound
h [Å]	400.0	1000.0
σ [Å]	0.01	10.0
σ_0 [Å]	0.01	50.0
ρ_1 [10^{-6} Å^{-2}]	3.01	8.0
ρ_0 [10^{-6} Å^{-2}]	2.07	2.07
r_{scale}	0.91	1.1
$\log_{10}$ background	-7.0	-4.3

Table S6: Parameters defining the layer of the one-layer `refnx` model parameters. Addition to Table S5.

Parameter	Value
h [Å]	860
σ [Å]	8
σ_0 [Å]	20
ρ_1 [10^{-6} Å^{-2}]	5.9
r_{scale}	1.0
$\log_{10}$ background	-6

Figure S3 shows the results for the d-PS/FBR sample measured during the temperature variation in the range of 334 – 402 K. Some of the curves were measured only at the first incident angle. Whenever the curves were additionally measured at the second incident angle both curves were concatenated. The prior bounds are in Table S8.

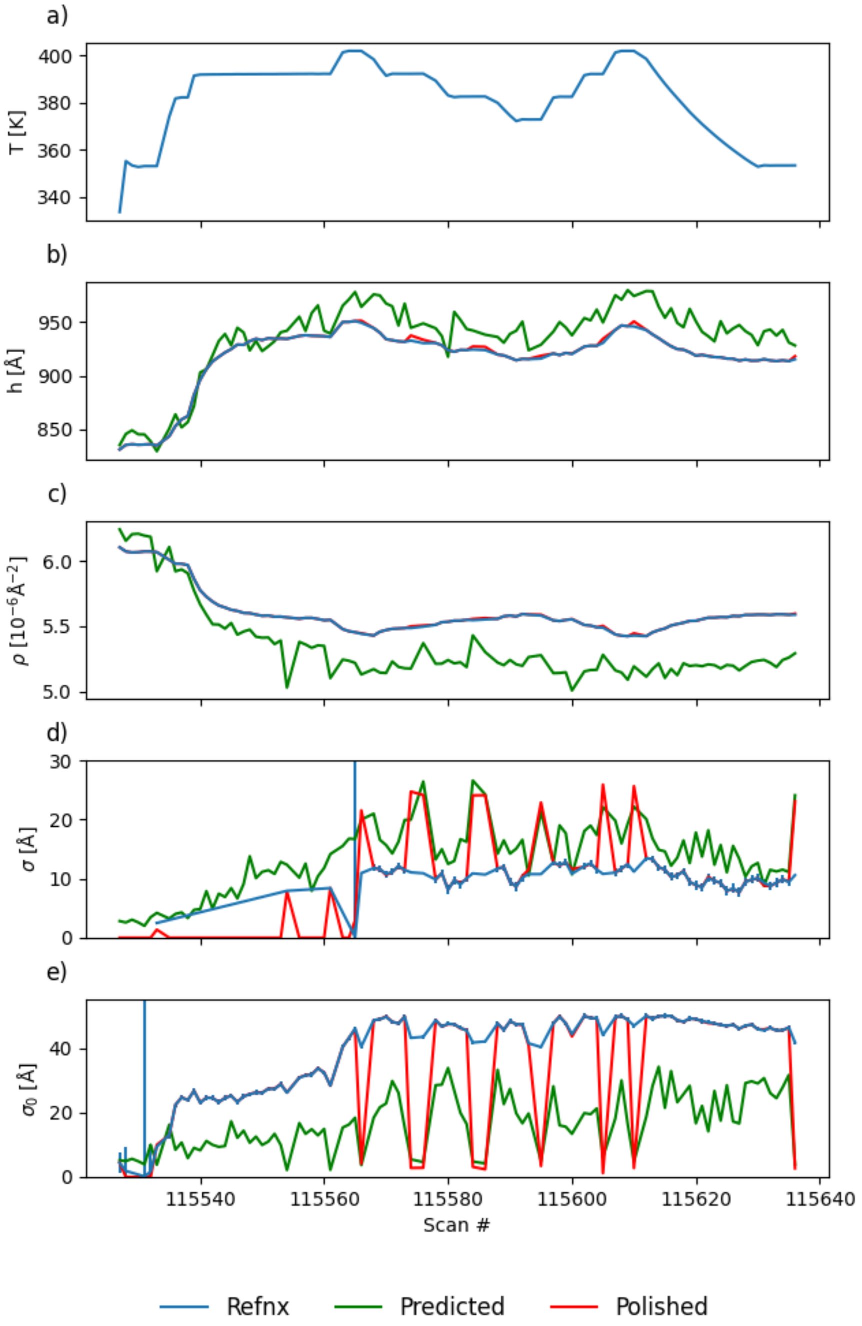

Figure S3: Variation of the sample parameters of d-PS/FBR during *in situ* annealing at different temperatures: temperature (a), layer thickness h (b), layer SLD ρ (c), layer roughness σ (d), substrate roughness σ_0 (e). The blue curves show the parameters extracted by `refnx`, the green curves show the raw predicted parameters provided by `reflectorch`, the results after the polishing step are in red. We omitted the error bars of the `refnx` fit where the value approached zero (lower boundary), as those parameter uncertainties are not meaningful. For clarity, we also omitted the uncertainties of the parameters obtained from the polished `reflectorch` predictions.

Table S7: Parameters defining the layer of the one-layer `refnx` model parameters for the *in situ* analysis, Figure S3, Addition to Table S8.

Parameter	Value
h [Å]	800
σ [Å]	10
σ_0 [Å]	15
ρ [10^{-6} Å^{-2}]	6
r_{scale}	1
$\log_{10}$ background	-6

Table S8: Prior bounds for the one-layer analysis during the *in situ* experiment for the `reflectorch` model and the `refnx` fit, the results are visualized in Figure S3.

Parameter	Lower bound	Upper bound
h [Å]	700.0	1000.0
σ [Å]	0.01	50.0
σ_0 [Å]	0.01	50.0
ρ [10^{-6} Å^{-2}]	4.0	7.0
ρ_0 [10^{-6} Å^{-2}]	2.07	2.07
r_{scale}	0.91	1.1
$\log_{10}$ background	-7.0	-4.3

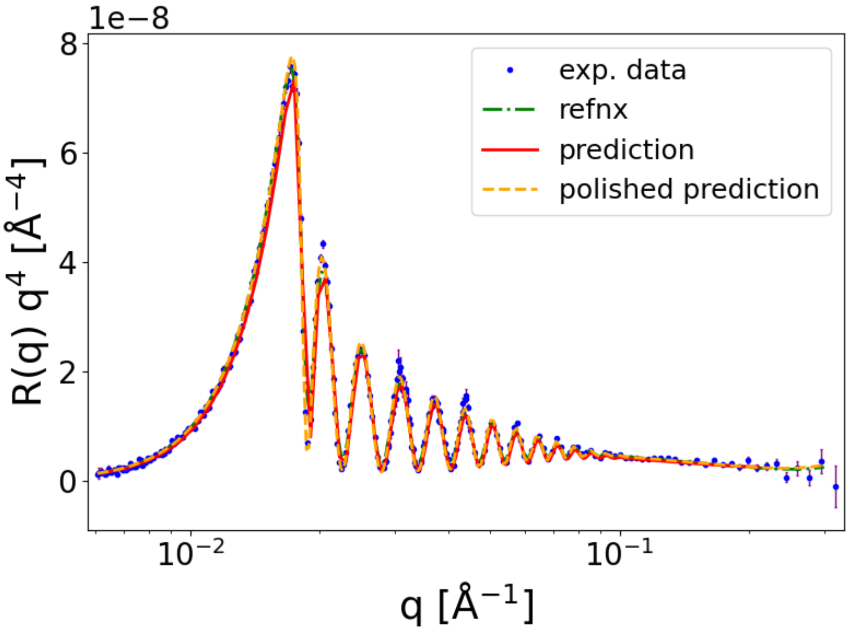

Figure S4: Fitting results for the d-PS/FBR sample. Addition to Figure 2(c).

S5 Additional information on sample preparation

The d-PS was supplied by Polymer Source Inc. (Canada) and the FBR and the bis-PCBM was supplied by Ossila (UK). The h-PS was supplied by Agilent (UK). The silicon substrates were supplied by Crystran (UK). The sheets of freshly cleaved mica were supplied by Goodfellow (UK). Samples were fabricated in the week before the NR experiment. The solution concentrations and spin-coater speeds used for each layer are in Table S9. After preparing the solutions, as described in Section 3, they were left in the dark overnight. The silicon substrates were sonicated in acetone and then isopropanol (15 minutes each). This was followed by rinsing in de-ionised water, and then by rotating the wafers at approximately 2000 rpm on a spin-coater for one minute. After depositing both layers on the silicon substrates, described in Section 3 and in the supporting information of (Higgins *et al.*, 2024), the samples were left in the dark, before being placed under vacuum (at room temperature, in the dark) for 24 hours. They were then stored for a few days in the dark in a glovebox prior to carrying out the experiment.

Table S9: Solution concentrations and spin-coater speeds used for the preparation of the two samples.

	solution concentrations [wt. %] (solid content)	spin-coater speed [rpm]
FBR	3	2200
d-PS	2.5	1900
bis-PCBM	2.5	2200
bis-PCBM/PS blend	1.6	1900

References

Glavic, A., Maranville, B., McCluskey, A. & Nelson, A. (2024). orsopy documentation. https://www.reflectometry.org/orsopy/. Copyright 2024, ORSO. Accessed: 2025-11-27.

Higgins, A., Gutfreund, P., Italia, V., Nelson, A., Cabral, J. & Hynes, E. (2024). *Soft Matter*, **20**, 2532–2546.

Munteanu, V., Starostin, V., Greco, A., Pithan, L., Gerlach, A., Hinderhofer, A., Kowarik, S. & Schreiber, F. (2024). *J. App. Cryst.* **57**, 456–469.

Nelson, A. R. & Prescott, S. W. (2019). *J. Appl. Crystallogr.* **52**, 193–200.
